# Health services utilization and out-of-pocket (OOP) expenditures in public and private facilities in Pakistan: an empirical analysis of the 2013–14 OOP health expenditure survey

**DOI:** 10.1186/s12913-021-06170-4

**Published:** 2021-02-25

**Authors:** F. Khalid, W. Raza, D. R. Hotchkiss, R. H. Soelaeman

**Affiliations:** 1 School of Public Health and Tropical Medicine, Tulane University, New Orleans, Louisiana USA; 2 Present address:Universal Health Coverage/Health Systems Department, World Health Organization, Regional Office for the Eastern Mediterranean, Monazamet El Seha El Alamia Str, Extension of Abdel Razak El Sanhouri Street, Nasr City, P.O. Box 7608, Cairo, 11371 Egypt; 3 Aga Khan University, Karachi, Pakistan; 4 Independent researcher, Atlanta, GA USA

**Keywords:** Out-of-pocket health expenditure, Healthcare utilization, Private sector, Universal health coverage, Pakistan

## Abstract

**Background:**

As low- and middle-income countries progress toward Universal Health Coverage, there is an increasing focus on measuring out-of-pocket (OOP) expenditure and health services utilization within countries. While there have been several reforms to improve health services coverage and financial protection in Pakistan, there is limited empirical research comparing OOP expenditure and health services utilization between public and private facilities and exploring their determinants, a knowledge gap addressed in this study.

**Methods:**

We used data from 2013 to 14 OOP Health Expenditure Survey, a population-based household survey carried out for Pakistan’s National Health Accounts. The analysis included 7969 encounters from 4293 households. We conducted bivariate analyses to describe patterns of care utilization, estimated annualized expenditures by type and sector of care, and assessed expenditure composition. We used multivariable logistic regression modeling to identify factors associated with sector of care and generalized linear model (GLM) with log link and gamma distribution to identify determinants of OOP expenditures stratified by type of care (inpatient and outpatient).

**Results:**

Most encounters (82.5%) were in the private sector and were for outpatient visits (85%). Several public-private differences were observed in annualized expenditures and expenditure components. Logistic regression results indicate males, wealthier individuals, Punjab and Sindh residents, and those in smaller households were more likely to access private outpatient care. In the inpatient model, rural residents were more likely to use a private provider, while Khyber Pakhtunkhwa residents were less likely to use private care. GLM results indicate private sector inpatient expenditures were approximately PKR 6660 (USD 61.8) higher than public sector expenditures, but no public-private differences were observed for outpatient expenditures. Several demographic factors were significantly associated with outpatient and inpatient expenditures. Of note, expenditures increased with increasing wealth, decreased with increasing household size, and differed by province and region.

**Conclusions:**

This is the first study comprehensively investigating how healthcare utilization and OOP expenditures vary by sector, type of care, and socio-economic characteristics in Pakistan. The findings are expected to be particularly useful for the next phase of social health protection programs and supply side reforms, as they highlight sub-populations with higher OOP and private sector utilization.

**Supplementary Information:**

The online version contains supplementary material available at 10.1186/s12913-021-06170-4.

## Background

As countries progress toward achieving Universal Health Coverage (UHC), there is an increasing focus on measuring and comparing both out-of-pocket (OOP) expenditures and health services utilization in low- and middle-income countries (LMICs) [1, 2]. Both utilization and OOP expenditures have strong links to coverage of essential health services and financial protection, the two types of indicators being used to track progress toward UHC [3]. Currently available global evidence shows that the share of total health expenditure that comes in the form of household OOP payments is relatively high across LMICs (over 50% on average) [4]. Similarly, research on health services utilization shows that private sector utilization is also relatively higher in LMICs compared to high-income countries, with over 50% of health services utilization in LMICs occurring at private facilities [1, 4]. However, further analysis of both OOP expenditures and utilization within countries and regions shows that there is also significant variation in these results between different socio-economic groups [5, 6]. The *Tracking Universal Health Coverage: 2017 Global Monitoring Report* acknowledges that there are variations in progress toward UHC within countries and encourages more in-depth country level analysis [3].

In Pakistan, a LMIC in South Asia and the sixth most populous country in the world, OOP expenditures account for 58% of the total health expenditure [7, 8]. A breakdown of the total OOP expenditure shows that 81% was spent in the private sector and 19% was incurred by users of public health facilities [8]. Pakistan has historically been a chronic underspender on healthcare; public spending on health makes less than 1% of its GDP and less than 5% of government expenditure is spent on health [8]. The National Health Accounts (NHA) report in 2015–16 shows that Pakistan is heavily reliant on the private sector for healthcare services, with approximately 85% of total health expenditure being incurred at private facilities, 58% of total health expenditure were in the form of OOP expenditures, 1% were private voluntary health insurance contributions, and 35% was government spending on health [8].

The federal and provincial health departments in Pakistan have undertaken several initiatives to reduce OOP expenditures and improve service delivery at both public and private facilities. For example, since 2015, the federal government and provincial governments of Khyber Pakhtunkhwa (KP) and Gilgit Baltistan established three major social health protection programs that provide fully subsidized health insurance coverage to households living below the poverty line (USD 2 a day). The programs are operational in over 100 districts across the provinces of KP, Punjab, and Gilgit Baltistan, and cover over 10 million households. Currently, all three major programs cover only inpatient expenses^1^ up to a certain threshold for specific illnesses.^2^ Each program has empanelled selected public and private hospitals to provide the services [9, 10]; details are presented in Additional file 1. The percentage of current health spending on the programs is not available in the NHA reports published so far. The programs were launched after the survey used for this study was completed.

Existing studies indicate that between 57 and 80% of health services utilization occurs in the private sector in Pakistan [11, 12]. For effective use of private sector for service delivery, the provincial governments of KP and Sindh have been promoting the use of public-private partnerships (PPP) by establishing PPP departments. The use of contracted firms from the private sector to manage public sector primary healthcare facilities is particularly dominant in the province of Sindh [13].

While there have been several reforms to improve health services coverage and financial protection in Pakistan, there is limited empirical research available comparing health services utilization and OOP expenditures between public and private facilities and exploring their determinants. The NHA report provided an estimate of the overall percentage of private and public sector health utilization, but did not provide disaggregation of utilization and OOP expenditures by type of provider and care accessed, disease categories, and socio-economic characteristics [8, 14]. Other studies have explored the determinants of public versus private sector health utilization in Pakistan but focused on a specific region or health issues [12, 15–17]. There have been no nationally representative studies comparing OOP expenditures and health care utilization across public and private sector facilities and exploring their determinants.

In this paper, we address this gap by using the 2013–2014 OOP Health Expenditure Survey to provide a comprehensive analysis of the determinants of health services utilization and OOP expenditures in public and private facilities across Pakistan for both inpatient and outpatient services across socio-economic groups and disease categories. We believe this analysis will be a useful and timely resource for policymakers and practitioners engaged with health financing and service delivery reforms, as provincial and federal health departments plan to scale up the social health protection programs to the national level, enrolling over 15 million poorest families (over 80 million individuals), and empanelling hundreds of health care facilities across the country [18, 19]. This analysis provides evidence that can be used to help guide these programs on the populations and disease burdens to target. Additionally, given the lack of costing studies available in Pakistan, the OOP expenditures reported by disease category, provider type and care accessed may be a useful point of reference for social protection programs.

## Methodology

### Data

This analysis is based on data from the 2013–14 OOP Health Expenditure Survey, a population-based household survey conducted for Pakistan’s National Health Accounts [14]. The OOP Health Expenditure Survey was conducted on a sub-sample of the larger Household Integrated Economic Survey (HIES) carried out by the Pakistan Bureau of Statistics. A sample of 4828 households was drawn using two-stage stratified random sampling with enumeration blocks selected at the first stage and households within the enumeration blocks selected at the second stage. Urban/rural status, as defined by the respective provincial governments, was used as sampling strata. Respondents in the sampled households were asked whether any household member had an illness within 1 year of the interview. Using the household roster, respondents were then asked questions about treatment-seeking behavior and expenditures for the person who was reported to have had an illness. Of the 4828 households sampled, 4293 (88.9%) reported at least one household member who had used health care services within the past year and had completed the survey. Information was not available for 535 (11.1%) households where no members accessed health care services within 1 year of the interview.

The survey contained questions on the type of healthcare provider accessed, type of illness, type of care accessed, and health expenditures for each care-seeking encounter over the recall period (3 months for outpatient care and 1 year for inpatient care). The questionnaire only allowed for the recording of one inpatient visit and one outpatient visit per household member. In all, there were 9021 encounters from 8895 unique household members who had an illness within 1 year of the interview date. Because we were interested in examining the determinants of the sector and OOP expenditures among those who sought formal medical care (defined as having an inpatient or outpatient care visit), we excluded encounters related to self-medication (*n* = 999 or 11.1% of the sample) and health facility or provider visits that were not related to an illness (*n* = 52 or 0.59%). The final analytic dataset contained information on 7969 encounters from 7863 individuals who sought medical care.

### Variables

The goal of this study was to investigate factors associated with sector of care and OOP expenditures among individuals who sought medical care. We examined two outcome variables in the analysis. The first outcome variable, the sector where care was sought, is a binary variable coded as 1 if the encounter occurred at a private facility or provider and 0 if the encounter occurred at a public sector facility or provider. Private sector providers include private hospitals, private physician clinics, traditional practitioners/healers, pharmacies/shops, and private laboratories. Government-owned facilities, including military hospitals, were classified as public sector providers. The second outcome variable was the amount paid out-of-pocket for medical care, a continuous variable. This variable included direct medical expenditures (doctors’ fees, and the cost of medicines and vaccines, diagnostic tests, surgery, and durables) and indirect expenditures (transportation costs, admission fees, food costs, tips, and the cost to the accompanying person). Model covariates included household- and individual-level characteristics, such as gender, age, wealth, region, province, household size, and type of illness. The type of illness variable was created by collapsing the disease type variable into the following categories: communicable, accident/injury, chronic, childbirth, and other female reproductive health concerns. Illnesses that did not fall under one of these categories were classified as “Other.”

### Statistical analysis

We conducted bivariate analyses to characterize the study sample and estimated unadjusted OOP expenditures by illness type and socio-demographic characteristics. Outpatient utilization was analyzed for the three-month recall period, but outpatient OOP expenditure was annualized for the descriptive and multivariable analyses. In the bivariate analysis, we first compared type of healthcare provider accessed by the setting of care for the encounter. Next, we compared a breakdown of the average annual OOP expenditures for outpatient and inpatient visits by sector of care. Finally, we compared proportions of expenditure composition per encounter for inpatient and outpatient visits by sector of care.

We used logistic regression models to investigate factors associated with the sector where care was sought. We fitted separate models for inpatient combined with delivery care and outpatient care because the factors driving the choice of setting differ based on an individual’s perceived need. Next, we identified drivers of OOP expenditures stratified by type of care using multivariable generalized linear models (GLM) with a log link and gamma distribution. For the GLM, we also included sector of care (i.e., public vs. private) as a covariate, since we were interested in estimating differences in OOP expenditures by sector of care. Modelling the determinants of health care expenditures is challenging because indicators of health care expenditure often have distributions that are skewed with a large mass with zero expenditures [20]. In this sample, less than 1 % of encounters had zero health care expenditure, negating the need to fit a two-part model. However, the expenditures were highly positively skewed. Following the methods outlined by Deb and Norton (2018), we conducted a modified Park’s test after running the GLM to empirically test the relationship between the mean and variance [20]. The estimated value of λ was 2.3 for the inpatient model and 2.1 for the outpatient model indicating that the gamma distribution is appropriate for both models. We used Stata 14 SE (StataCorp, 2015) for data management and all analyses [21]. Estimates were weighted and standard errors clustered to account for the complex sampling design.

## Results

### Study sample characteristics

Characteristics of the study sample and their health encounters are shown in Table 1. Of the 7878 people who had an illness within 1 year of interview, the highest percentage of people who sought care were 41 to 60 years old for outpatient encounters (25.3%), and 21 to 40 years old for inpatient/delivery encounters (47.4%). When utilization was examined by household wealth, the highest percentage of people who sought care were in the wealthiest quintile for both inpatient (28.3%) and outpatient (22.5%) visits, while the lowest percentage was in the poorest quintile for both types of visits (16.3% for outpatient, 12.5% for inpatient). Approximately half of the respondents lived in Punjab (49.1% for outpatient, and 57.6% for inpatient), and most respondents lived in rural areas (61.8% for outpatient, and 69.3% for inpatient). Most (85.9%) of the care sought took place in an outpatient setting. The results suggest a preference for private providers for both outpatient and inpatient care (84.6 and 68.5%, respectively).

**Table 1:** Characteristics of the study sample and their health encounters

	Outpatient		Inpatient/ Delivery	
	n	%	n	%
**Characteristics of the study sample (*n*** **= 7878)**				
**Total**	6724	85.3	1154	14.6
**Age**				
0 to 5 years	1226	18.2	76	6.6
6 to 20 years	1632	24.3	143	12.4
21 to 40 years	1415	21.1	547	47.4
41 to 60 years	1701	25.3	248	21.5
> 60 years	749	11.1	140	12.1
**Gender**				
Male	2950	43.9	358	31
Female	3774	56.1	797	69
**Household wealth quintile**				
Poorest	1097	16.3	145	12.5
Poorer	1194	17.8	165	14.3
Middle	1408	20.9	232	20.1
Richer	1513	22.5	287	24.8
Richest	1512	22.5	327	28.3
**Province**				
Punjab	3300	49.1	665	57.6
Sindh	2384	35.5	252	21.8
KP	827	12.3	198	17.1
Balochistan	214	3.2	39	3.4
**Region**				
Urban	2567	38.2	355	30.7
Rural	4157	61.8	800	69.3
**Household size**				
1 to 4	1490	22.2	206	17.8
5 to 8	3688	54.9	604	52.3
9 to 12	1171	17.4	247	21.4
13+	375	5.6	98	8.5
**Characteristics of healthcare encounters (*n*** **= 7969)**				
**Total**	6770	85.9	1199	15
**Type of illness**				
Communicable	1108	16.4	150	12.5
Accident/Injury	112	1.7	71	5.9
Chronic	1705	25.2	237	19.8
Childbirth	N/A		450	37.5
Other female reproductive health concerns	319	4.7	55	4.6
Other	3525	52.1	237	19.7
**Sector of care**				
Public	1043	15.4	378	31.5
Private	5728	84.6	821	68.5

### Bivariate analysis

**Fig. 1:**
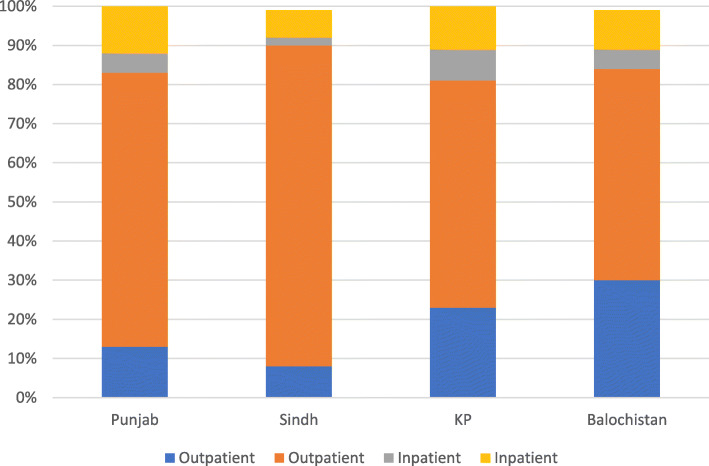
Type of provider accessed by province

**Fig. 2:**
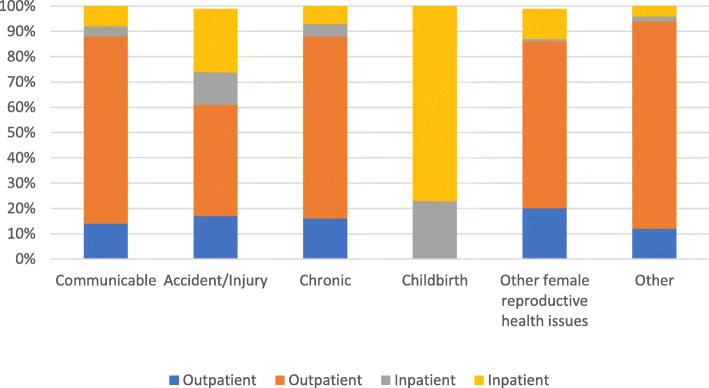
Type of provider accessed by disease category

As shown in Figs. 1 and 2, the province of residence and illness type were associated with care sector choice. Residents of Punjab and Sindh provinces had higher private sector outpatient care utilization at 70 and 82% respectively, compared to KP and Balochistan provinces, where this figure was much lower at 58 and 54%, respectively. Approximately 90% of utilization for communicable diseases and chronic conditions was for outpatient care, with over 70% of encounters occurring at private sector facilities. Accidents and injuries had a relatively higher percentage of outpatient utilization (61%) compared to inpatient utilization (39%). Approximately 52% of outpatient and 20% of inpatient encounters were for illnesses classified as “Other”, and over 80% of outpatient encounters for these illnesses were at private sector health facilities.

Table 2 presents the distribution of healthcare encounters by provider type and type of care. Most outpatient encounters occurred with a private doctor/clinic (69.4%), followed by public tertiary care (12%), and private hospitals (8.3%). Most inpatient encounters occurred at private hospitals (51.7%), public tertiary care hospitals (27.8%), and with a private doctor/clinic (8.3%). It is worth noting that traditional modes of care were also consulted; 5.3 and 7.5% of those who sought medical care chose traditional healers for outpatient and inpatient care, respectively.

**Table 2:** Type of healthcare provider accessed by type of care

	Type of care^**a,b**^	
	Outpatient	Inpatient
	(***n*** = 6770)	(***n*** = 1199)
**Public sector providers**		
Community health workers (Lady Health Visitor [LHV]/Nurse, Lady Health Worker [LHW])	0.1	1.2
Primary care (Basic Health Unit [BHU], Rural Health Center (RHC), Dispensary/Maternal & Child Health Care Center)	2.7	0.8
Secondary care (Tehsil Headquarter [THQ]/District Headquarter Hospital [DHQ])	0.2	0.8
Tertiary care (Tertiary, teaching or specialized hospital, Government hospital)	12.0	27.8
Autonomous bodies/semi-government hospital (Military Hospital, Social Security Hospital, & other autonomous bodies)	0.4	0.9
***Public sector provider sub-total***	***15.4***	***31.5***
**Private sector providers**		
Private Doctor/Clinic	69.4	8.0
Private Hospital	8.3	51.7
Traditional modes of care (Homeopath/Hakeem/ Herbalist/Saina /Dai)	5.3	7.5
Others (Laboratory, Pharmacy/Shops, & other private facilities)	1.5	1.4
***Private sector provider sub-total***	***84.6***	***68.5***
**Total**	**100.0**	**100.0**

^a^Column percentages shown

^b^Chi-squared test *p* < 0.001

Table 3 shows average annualized expenditure (in 2013 PKR)^3^ for outpatient and inpatient care by sector of care. Although the average outpatient expenditures in the public and private sectors were similar (PKR 10,440 in the public sector and 10,395 in the private sector), there were differences in individual expenditure components. For example, outpatient expenditures on doctors’ fees were significantly higher (*p* < 0.001) in the private sector, where the mean expenditure on doctors’ fees was PKR 2110 compared to PKR 29 in the public sector. Expenditures on supplies and medical durables were also higher in the private sector than in the public sector (PKR 550 vs. PKR 300, respectively, *p* = 0.04). Other categories of medical expenditures for outpatient care, such as spending on medicines and diagnostic tests, were not significantly different between the public and private sectors. Like outpatient care, the average inpatient expenditures in the public and private sectors were similar, but differences were observed when expenditure components were examined. Private sector inpatient encounters led to significantly higher expenses on the following categories of medical expenditures: parchi/admission fee, doctors’ fees, and operation theater/intervention room charges (all *p*-values < 0.001). The average expenditure on inpatient admission fees was PKR 889 in the private sector compared to PKR 63 in the public sector. Expenditures on doctors’ fees and operation theater expenses were PKR 3646 and PKR 3294, respectively, in the private sector compared to PKR 581 and PKR 883, respectively, in the public sector. Among non-medical expenditures for inpatient encounters, expenditure on tips was significantly higher in the private sector (PKR 74) than in the public sector (PKR 35, *p* = 0.01).

**Table 3:** Average annualized expenditure (PKR) for outpatient and inpatient by sector of care

	Outpatient				***p***-value	Inpatient				***p***-value
	Public sector (***n*** = 1043)		Private sector (***n*** = 5728)			Public sector (***n*** = 378)		Private sector (***n*** = 821)		
	Mean	(SD)	Mean	(SD)		Mean	(SD)	Mean	(SD)	
**Medical**										
Parchi and admission fee	43.81	(149.84)	61.92	(639.60)	0.07	63.24	(674.90)	889.00	(2311.63)	< 0.001
Medicine/ vaccine	7877.19	(26,167.09)	5399.12	(10,427.44)	0.11	3438.94	(61,366.47)	7114.99	(16,515.53)	0.51
Supplies/ medical durables	300.56	(1520.27)	550.12	(5556.40)	0.04	4065.80	(60,948.26)	1213.20	(5583.05)	0.40
Diagnostic tests	1008.59	(3721.70)	1059.85	(3823.73)	0.73	2182.43	(7616.12)	1610.13	(4441.35)	0.22
Doctors’ fee	28.72	(407.31)	2109.64	(3555.00)	< 0.001	580.78	(6302.82)	3646.50	(5290.76)	< 0.001
Operation theater/ intervention room charges	N/A		44.97	(1318.50)		883.46	(5960.55)	3294.18	(14,471.24)	< 0.001
**Non-medical**										
Food	162.35	(1045.61)	164.30	(624.13)	0.96	587.86	(1231.55)	608.03	(1142.96)	0.82
Tips	5.57	(144.49)	3.89	(87.66)	0.72	34.99	(159.76)	73.56	(292.25)	0.01
Transportation	863.50	(2072.86)	829.05	(2651.37)	0.69	1000.15	(3142.08)	831.80	(1258.59)	0.39
Accompanying person costs	97.73	(669.64)	52.88	(462.90)	0.27	747.29	(3101.04)	559.51	(1560.77)	0.35
Other	52.55	(373.22)	119.55	(553.66)	< 0.001	95.03	(252.56)	76.19	(229.09)	0.22
**Total**	**10,440.56**	**(29,456.01)**	**10,395.29**	**(18,248.51)**	**0.98**	**19,694.94**	**(135,422.50)**	**19,917.08**	**(34,109.45)**	**0.98**

Table 4 shows the differences in expenditure composition per visit or admission by type and sector of care. In the outpatient setting, medicines and vaccines accounted for about three quarters of public sector OOP expenditures (75.4%); other major drivers of public sector OOP expenditures were diagnostic tests (9.7%) and transportation (8.3%). Medicines and vaccines were also major drivers of expenditures for private sector outpatient visits, but their share of total OOP expenditures (51.9%) was not as large as in the public sector. Instead, doctors’ fees (20.3%) and diagnostic tests (10.2%) collectively accounted for almost 31% of all private sector outpatient OOP expenditures. We observed similar patterns for inpatient care: public sector expenditures were driven by medicines and vaccines (48%), supplies and medical durables (20.6%), and diagnostic tests (11.1%), while private sector OOP expenditures were driven by medicines and vaccines (35.7%), doctors’ fees (18.3%), and operation theater or room charges (16.5%).

**Table 4:** Expenditure composition per visit or admission by type and sector of care

	Outpatient^**a**^		Inpatient^**a**^	
	Public sector	Private sector	Public sector	Private sector
	(***n*** = 1043)	(***n*** = 5728)	(***n*** = 378)	(***n*** = 821)
**Medical**				
Parchi and admission fee	0.4	0.6	0.3	4.5
Medicine/vaccine	75.4	51.9	48.0	35.7
Supplies/medical durables	2.9	5.3	20.6	6.1
Diagnostic tests	9.7	10.2	11.1	8.1
Doctors’ fee	0.3	20.3	2.9	18.3
Operation theater/ intervention room charges	N/A	0.4	4.5	16.5
**Non-medical**				
Food	1.6	1.6	3.0	3.1
Tips	0.1	0.0	0.2	0.4
Transportation	8.3	8.0	5.1	4.2
Accompanying person costs	0.9	0.5	3.8	2.8
Other	0.5	1.2	0.5	0.4
**Total costs**	**100.0**	**100.0**	**100.0**	**100.0**

^a^Column percentages shown

### Multivariable models

#### Factors associated with sector of care

We examined the factors associated with the sector where care was sought among those who sought medical care. Because we anticipated that the factors would differ for those who sought inpatient care or delivery assistance compared to those who sought outpatient care, we analyzed the data on sector of care separately based on the type of care. The regression results are presented in Table 5.

**Table 5:** Marginal effects from logistic regression models: factors associated with choosing a private sector provider vs. a public sector provider stratified by type of care

Pr(Private)	Outpatient			Inpatient		
	Marginal effect	SE	***p***-value	Marginal effect	SE	***p***-value
Gender						
Male	Ref.			Ref.		
Female	−0.025	0.01	0.02	0.034	0.04	0.41
Age						
0 to 5	Ref.			Ref.		
6 to 20	−0.003	0.02	0.88	−0.042	0.07	0.52
21 to 40	−0.005	0.02	0.78	−0.018	0.06	0.78
41 to 60	−0.016	0.02	0.37	−0.096	0.07	0.14
> 60	− 0.011	0.02	0.62	−0.024	0.07	0.73
Household wealth quintile						
Poorest	Ref.			Ref.		
Poorer	0.001	0.02	0.97	−0.010	0.07	0.87
Middle	0.002	0.02	0.93	0.058	0.06	0.34
Richer	0.058	0.02	0.001	0.004	0.06	0.95
Richest	0.075	0.02	< 0.001	0.082	0.06	0.19
Region						
Rural	0.001	0.01	0.92	0.112	0.04	0.001
Province						
Punjab	Ref.			Ref.		
Sindh	0.062	0.01	< 0.001	0.053	0.04	0.14
KP	−0.133	0.02	< 0.001	−0.185	0.04	< 0.001
Balochistan	− 0.187	0.03	< 0.001	−0.065	0.06	0.30
Household size						
1–4	Ref.			Ref.		
5–8	−0.050	0.01	< 0.001	0.028	0.04	0.53
9–12	−0.051	0.02	0.001	−0.033	0.05	0.54
13+	−0.060	0.03	0.03	−0.026	0.07	0.72
Type of illness						
Communicable	Ref.			Ref.		
Accident/Injury	−0.107	0.05	0.04	0.005	0.08	0.95
Chronic	−0.014	0.02	0.40	−0.095	0.06	0.13
Childbirth	N/A			0.056	0.06	0.38
Other female reproductive health concerns	−0.053	0.03	0.08	0.159	0.08	0.05
Other illness	0.033	0.01	0.02	−0.008	0.06	0.89
*N*	*6770*			*1199*		
*Pseudo R-squared*	*0.06*			*0.06*		

Among patients who accessed outpatient care (*n* = 6724), females were 2.5 percentage points (pp) less likely than males to choose care from a private sector provider (*p* = 0.02). In addition, the choice of sector also appeared to follow a wealth gradient, where patients in the richest quintile was 7.5 pp. (*p* < 0.001) more likely, and the richer quintile 5.8 pp. (*p* = 0.001) more likely than those in the poorest quintile to choose private sector care. There were no statistically significant differences in sector of care for patients in middle and poor wealth quintiles compared to the poorest. We also observed that the likelihood of seeking outpatient care from the private sector was negatively correlated with household size. Those living in households with more than four members were less likely to choose private sector providers than those in households with one to four members (marginal effect range − 6.0 pp. to − 5.0 pp., all *p*-values < 0.05). Finally, we found that private sector care was more likely to be sought for some illness types than others. For example, compared to communicable diseases, accidents and injuries were less likely to be treated in the private sector, whereas other illnesses were more likely to be treated in the private sector.

The patterns observed among those who accessed outpatient care were quite different from the patterns observed among those who accessed inpatient and delivery care. Notably, in contrast to those who accessed outpatient care, gender, wealth, and household size were not associated with sector of care. In addition, patients living in a rural region who accessed inpatient care were 11.8 percentage points more likely to seek private care than those in urban areas (*p* = 0.001). Finally, the only illness type that was significantly associated with seeking private inpatient care was other female reproductive health concerns (marginal effect 18.4 pp., *p* = 0.01). No statistically significant differences were observed for the remaining illness type categories.

#### Factors associated with out-of-pocket (OOP) expenditures

The results from the GLM on OOP expenditures stratified by type of care accessed are presented in Table 6. Among individuals who accessed outpatient care, patient age was positively associated with OOP expenditures and appeared to follow a gradient with increasing age. Compared to patients age 0 to 5 years, expenditures for patients age 6 to 20 years were, on average, PKR 1750 higher, expenditures for patients age 21 to 40 years PKR 5033 higher, expenditures for patients, age 41 to 60 years PKR 8093 higher, and expenditures for patients over 60 years old PKR 7374 higher (all *p*-values ≤0.001). OOP expenditures for outpatient care differed by wealth quintiles. Compared to the poorest wealth quintile, patients in the poorer, middle, richer, and richest quintiles spent PKR 1573, PKR 3387, PKR 4335, and PKR 7302 more on outpatient care (all *p*-values ≤0.001). Rural residents spent PKR 1455 more than their urban counterparts (*p* = 0.01). Additionally, compared to people living in Punjab province, people who lived in Sindh and KP provinces spent less, on average, for outpatient care (PKR 4665 and PKR 4394 respectively, both *p*-values < 0.001). People in the largest households (13 or more members) spent PKR 3388 less on average than those in households with 1 to 4 members (*p* = 0.01). There were no differences in OOP expenditures between private and public sector care.

**Table 6:** Factors associated with OOP expenditures stratified by type of care

OOP Expenditure^a^	Outpatient			Inpatient		
	Marginal effect	SE	***p***-value	Marginal effect	SE	***p***-value
Sector of care						
Private	1302.64	1179.10	0.27	6656.55	1359.37	< 0.001
Gender						
Female	−669.61	687.30	0.33	−4636.28	2330.86	0.05
Age						
0 to 5	Ref.			Ref.		
6 to 20	1749.97	530.25	0.001	4647.41	2554.70	0.07
21 to 40	5033.45	685.47	< 0.001	11,412.02	3626.46	0.002
41 to 60	8093.58	1134.86	< 0.001	4098.84	2401.64	0.09
> 60	7374.50	1174.63	< 0.001	3207.23	2764.51	0.25
Household wealth quintile						
Poorest	Ref.			Ref.		
Poorer	1573.21	613.17	0.01	1536.80	1235.42	0.21
Middle	3387.03	1048.08	0.001	4470.90	1298.43	0.001
Richer	4334.84	787.13	< 0.001	7423.11	1641.75	< 0.001
Richest	7302.04	1230.81	< 0.001	22,536.90	3227.64	< 0.001
Region						
Rural	1454.51	591.75	0.01	3296.88	1479.93	0.03
Province						
Punjab	Ref.			Ref.		
Sindh	− 4664.80	575.07	< 0.001	−3136.24	1450.22	0.03
KP	− 4393.93	750.38	< 0.001	447.40	2324.60	0.85
Balochistan	− 1621.27	1020.91	0.11	− 8523.96	1637.83	< 0.001
Household size						
1–4	Ref.			Ref.		
5–8	− 1183.92	721.30	0.10	−5581.07	2299.34	0.02
9–12	− 1833.67	951.51	0.05	−9167.43	2577.53	< 0.001
13+	−3388.54	947.73	< 0.001	1127.79	6448.54	0.86
Type of illness						
Communicable	Ref.			Ref.		
Accident/Injury	3397.88	2521.68	0.18	13,214.49	4524.63	0.003
Chronic	1442.92	919.07	0.12	4133.48	2739.33	0.13
Childbirth	N/A			− 3683.05	2423.11	0.13
Other female reproductive health concerns	760.92	1433.90	0.60	− 2495.16	3035.34	0.41
Other illness	− 4268.83	679.27	< 0.001	13,546.45	4022.53	0.001
*N*	*6770*			*1199*		

^a^Modeled using GLM with log link and gamma distribution, marginal effects are shown

Among individuals who accessed inpatient care, we found that OOP expenditures on private sector provider were PKR 6657 higher than expenditures on public sector providers (*p* < 0.001). Expenditures for females were PKR 4636 less than for males (*p* = 0.05). Compared to patients age 0 to 5 years, OOP expenditures were only significantly different among patients age 21 to 40 years (PKR 11,412, *p* = 0.002). Similar to expenditures on outpatient care, expenditures on inpatient care followed a wealth gradient. Compared to the poorest wealth quintile, patients in the middle, richer, and richest quintiles spent PKR 4471, PKR 7423, and PKR 22,537 more on inpatient care (all *p*-values ≤0.001). Geography was also associated with expenditures in the inpatient care model. Rural residents spent PKR 3297 more than their urban counterparts (*p* = 0.03), while residents of Sindh and Balochistan spent PKR 3136 and PKR 8524 less than Punjab residents (both *p*-values < 0.05). Household size was also associated with OOP expenditures. Households with 5 to 8 members and 9 to 12 members spent PKR 5581 and PKR 9167 less than households with 1 to 4 members, respectively (both *p*-values < 0.05). Finally, expenditures for inpatient care related to accident or injury was PKR 13,215 higher than for inpatient care related to communicable diseases (*p* = 0.003).

## Discussion

This is the first study to comprehensively investigate how healthcare utilization and OOP expenditures differed by sector, type of care, and socio-economic characteristics in Pakistan. Its findings will be useful for the federal and provincial health ministries in planning and monitoring the impact of the next phase of their social health protection programs and initiatives for public-private partnerships in the health sector. This study adds to the limited evidence base of research in LMICs for gauging disparities in healthcare utilization and OOP expenditures across different population groups. The use of data from the National Health Accounts OOP Health Expenditure Survey enables use of these findings for future research both within Pakistan and across LMICs.

An important finding from our study is the high utilization of private sector providers (82.2% of all encounters), a finding also reported in previous studies from Pakistan, including the Demographic and Health Survey [11, 15–17]. Globally, there is a growing consensus about importance of engaging with the private sector for achieving UHC due to the high utilization of private sector facilities in LMICs [2, 21]. The global evidence on the benefits of engaging the private sector in countries with high private sector utilization and our province-specific findings on the high utilization of private sector in Punjab and Sindh may provide a rationale for reforms related to public-private partnerships in healthcare.

The goal of UHC is to ensure that individuals and communities can access health services that they need without risk of financial hardship. A key finding from our study is that both poorer and larger households are spending less compared to than their richer and smaller household counterparts. On average, the wealthiest quintile spent nearly PKR.7400 more for outpatient care, and over PKR 22,500 more for inpatient care compared to the poorest quintile. Larger households spent significantly less than smaller households for outpatient care. Households with 9 to 12 members spent approximately PKR 1800 less and households with 13 or more members spent approximately PKR 3400 less than households with 1 to 4 members.

The difference in OOP expenditures between wealth quintiles and household size suggests that poorer families and larger families are either accessing poorer quality healthcare or forgoing expensive care and may have significant unmet need due to financial constraints. This interpretation of our results on the effect of wealth validates the bottom-up approach adopted by the government that targets the programs to the poorest segment of the population [9, 10]. It also supports the programs’ decision to enroll all members of the household regardless of family size. In addition to differences by wealth quintile and household size, we also observed differences by province. We also found geographic disparities in expenditures. Residents of Punjab spent more on outpatient care than residents of Sindh and KP, and more on inpatient care than residents of Sindh and Balochistan. These differences may be due to a combination of differences in quality of care across the provinces and unmet need. Additional research is needed on unmet need for medical care, including the populations most likely to forgo care, as well as diseases for which care is less likely to be sought (i.e., due to costs), as this information would be greatly useful for social health protection programs to design an appropriate benefit package.

This study has yielded other important findings for social health protection programs. For instance, we observed that not only do higher percentages of encounters across provinces and disease types occur in the private sector, private sector expenditures were also higher than public sector expenditures for inpatient care by PKR 6657, on average, a finding that supports the strategy used by social protection programs to empanel private sector hospitals.

The detailed analyses examining the differences in annualized expenditures and expenditure composition for both public and private sector providers for inpatient care suggest that social health protection programs have appropriately selected the expenditure categories that are most important for financial risk protection (i.e., doctors’ consultation fees, admission, medicines, supplies and medical durables, diagnostic tests, operation theater/intervention room, and transportation fees). However, each program has an annual coverage limit, and future research should explore the extent to which programs have been able to provide effective financial protection for inpatient needs.

Further, based on our findings, we recommend that any expansion in the benefit package of social health protection programs should include outpatient care, as 85.3% of all care encounters occurred in the outpatient setting, including close to 90% of encounters due to communicable diseases and chronic conditions. The current benefit packages include coverage for inpatient care,^4^ which may not be sufficient to provide adequate financial protection. Our findings regarding outpatient utilization and OOP expenditures, especially those related to geographic location, household size, and wealth status, can be used for developing appropriate payment mechanisms for strategically purchasing outpatient care.

Similar to findings from other LMICs, consultation fees (usually including doctors’ and paramedics’ fees, facility visit, or admission charges) were not found to be among the main expenditure drivers for both outpatient and inpatient care in public facilities, although they accounted for at least 20% of expenditures at private facilities [4]. Future reforms should go beyond the abolishment of user fees to include the provision of essential services, including supply of medicines, medical durables, and diagnostics at public facilities. Coverage for private sector care should include user fees in addition to provision of essential services. In addition, we observed that 5.3% of outpatient encounters and 7.5% of inpatient encounters occurred at traditional practitioners/healers. Traditional health care providers can be a cost-effective source of care for patients, but studies from Pakistan suggest that further efforts may be needed to integrate and regulate traditional healers by setting up the relevant governing bodies and research institutions, and regulating prescription and care practices [22, 23]. This recommendation is similar to findings regarding the role of traditional care from the region, other LMICs and global recommendations [24, 25].

Our study also shows three important results that should be explored for further research. The timing of our study coincides with contracting in reforms in the province of Sindh, where currently all public primary care facilities have been contracted out to private providers. Our analysis shows that 89% of encounters in Sindh used private sector facilities, and it may be helpful to evaluate the impact of contracting on OOP expenditures and public sector utilization through a follow-up survey. Another interesting finding from our analysis is that rural residents were 11.2 pp. more likely to visit private sector providers for inpatient care than urban residents. Investigating the reason for private sector preference in rural areas, including the presence of public facilities and residents’ perceived quality of care, could be an important area for further research. Finally, this study analyzed the determinants of OOP expenditures and private sector utilization among those who sought medical care. Additional research on determinants of self-medication or forgoing care among those who are sick may be instructive in designing programs, policies, or communications campaigns to increase demand generation for medical care.

The findings of this study are subject to a number of limitations. First, this analysis used only one wave of the NHA data, as the different recall periods for other waves preclude comparing trends in OOP expenditures over time. Our findings, therefore, represent a snapshot in time. Second, because the recall period for outpatient care was only 3 months, it is not possible to investigate annual outpatient utilization rates. Instead, we could only estimate the percentage of care sought in either the public or private sectors among those who sought outpatient care during the recall period. Third, our ability to explore the reasons for utilizing healthcare is limited, as over 50% of outpatient and nearly 20% of inpatient encounters were due to numerous “other” illnesses that were too small to present on their own; other categories, such as “women’s issues,” were poorly labelled. As a result, we could only make limited conclusions on the disease-specific utilization and related OOP expenditures. Fourth, the survey collected only basic information, so other variables that may affect selection of care sector, such as health insurance coverage, could not be controlled for in the models. In addition, the dataset only included households where at least one member reported an illness in the 12 months, so it was not possible to use a Heckman-type correction to account for potential sample selectivity. Finally, this analysis was based on data collected prior to the implementation of social health protection programs in Pakistan. The results presented for OOP expenditures and expenditure composition for inpatient care may have changed after the implementation of the programs. However, we do not expect these reforms to have had a large impact on the overall results since most of the utilization and OOP expenditures were for outpatient care, which is not covered in the social health protection programs. An important area of future research is to evaluate the impact of these programs using updated OOP expenditures survey data, which is expected to be published by Pakistan Bureau of Statistics in early 2021.

## Conclusion

In this study, we comprehensively analyzed a nationally representative dataset to fill the knowledge gap in Pakistan-specific research on health services utilization and OOP expenditures. The findings provide baseline estimates for OOP expenditures and their determinants before social health protection reforms were enacted. Federal and provincial governments, Pakistan Bureau of Statistics, and related development partners should develop a consensus on the type of evidence to be generated through regular surveys for gauging the impact of social health protection programs on service coverage and financial protection of the enrolled population The non-negligible percentage of both outpatient and inpatient encounters occurring at traditional practitioners and healers highlight the need for the government to bolster its efforts for bringing these into the national dialogue. Private sector engagement must be carefully managed to ensure financial protection of individuals who seek care.

## Supplementary Information

**Additional file 1.** Features of Pakistan’s three main social health protection programs.

## Data Availability

The data used in this study were obtained from National Health Accounts, Pakistan Bureau of Statistics, but restrictions apply to the availability of these data, which were used under license for the current study, and so are not publicly available. However, data are available from the authors upon reasonable request and with permission of the Pakistan Bureau of Statistics.

## Abbreviations

HIES: Household Integrated Economic Survey
KP: Khyber Pakhtunkhwa
LMIC: Low- and middle-income country
NHA: National Health Accounts
OOP: Out-of-pocket
UHC: Universal Health Coverage

## Footnotes

1 With the exception of outpatient visits for follow-up care after discharge from the health facility

2 More details on the diseases covered and maximum thresholds for each program can be found in the annex

3 The exchange rate at the time was 1 USD = 106.8 PKR

4 With the exception of outpatient visits for follow-up care after discharge from the health facility
