# Correction to: Health services utilization and out-ofpocket (OOP) expenditures in public and private facilities in Pakistan: an empirical analysis of the 2013–14 OOP health expenditure survey

**DOI:** 10.1186/s12913-021-06545-7

**Published:** 2021-06-10

**Authors:** Faraz Khalid, Wajeeha Raza, David R. Hotchkiss, Rieza H. Soelaeman

**Affiliations:** 1 School of Public Health and Tropical Medicine, Tulane University, New Orleans, Louisiana USA; 2 Present address: Universal Health Coverage/Health Systems Department, World Health Organization, Regional Office for the Eastern Mediterranean, Monazamet El Seha El Alamia Str, Extension of Abdel Razak El Sanhouri Street, P.O. Box 7608, Nasr City, Cairo 11371 Egypt; 3 Aga Khan University, Karachi, Pakistan; 4 Independent researcher, Atlanta, GA USA

**Correction to: BMC Health Serv Res 21, 178 (2021)**

**https://doi.org/10.1186/s12913-021-06170-4**

Following the publication of the original article [1], the authors identified several correction.

1. The legends for Figs. 1 and 2 do not show the full name of the values. The values should be ‘outpatient-private, outpatient public, inpatient-public and inpatient-private’. The correct figures are given below.

**Fig. 1:**
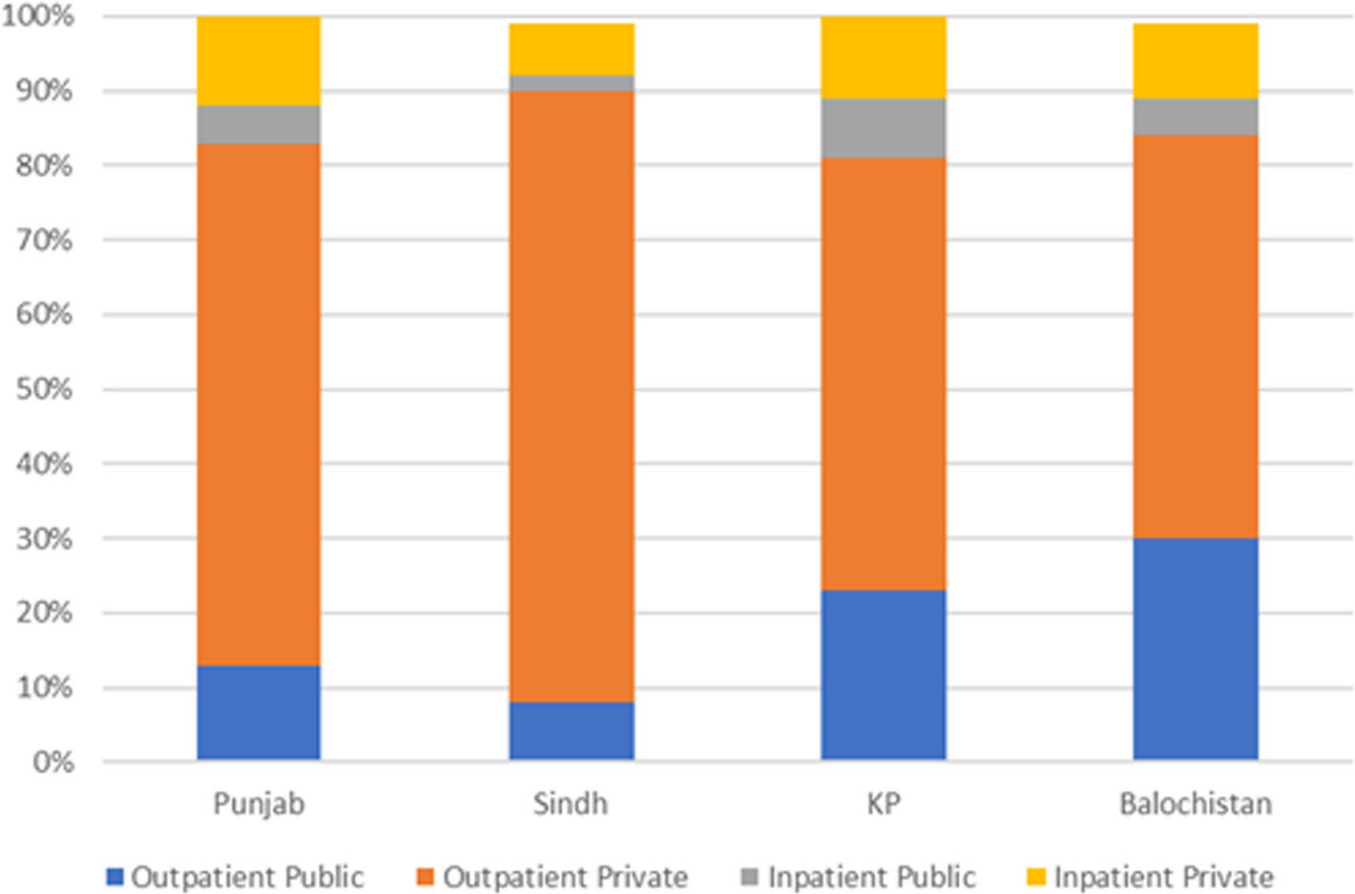
Type of provider accessed by province

**Fig. 2:**
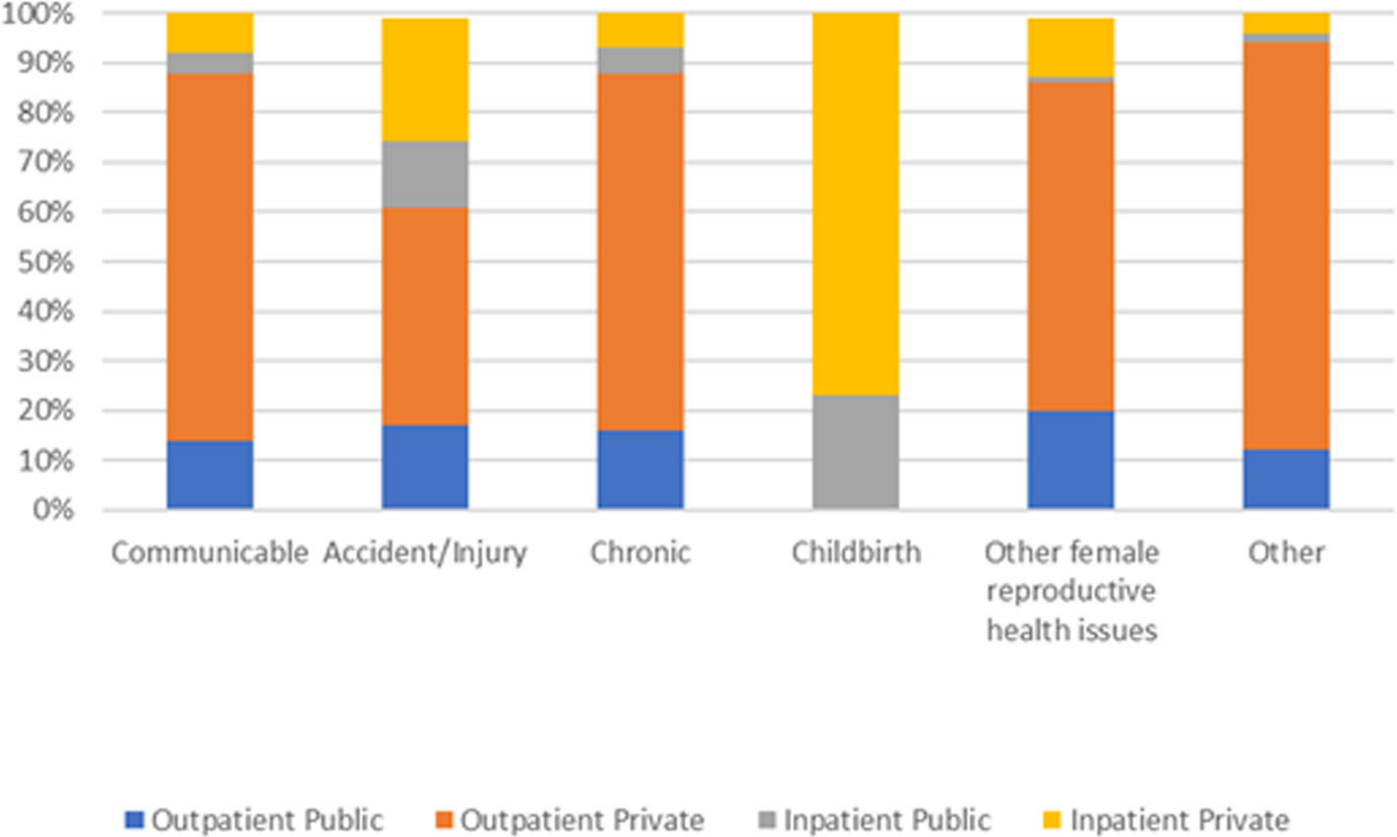
Type of provider accessed by disease category

2. The first name for all authors is written as an abbreviation, but the authors want to change the author names to the full names:

Faraz Khalid, Wajeeha Raza, David R. Hotchkiss, Rieza H. Soelaeman.

3. The second footnote on the page 2 should read “More details on the diseases covered and maximum thresholds for each program can be found in the additional file”. It currently refers to “in the annex” instead of “in the additional file”.
