# Supplementary material for: Health services utilization and out-of-pocket (OOP) expenditures in public and private facilities in Pakistan: an empirical analysis of the 2013–14 OOP health expenditure survey
Source: BMC Health Serv Res. 2021 Feb 25;21:178. doi: 10.1186/s12913-021-06170-4 (PMC7905921; doi:10.1186/s12913-021-06170-4)
Supplement: Supplementary file 1 — Additional file 1. Features of Pakistan’s three main social health protection programs. [file 12913_2021_6170_MOESM1_ESM.docx]

**Annex 1: Features of Pakistan’s three main social health protection programs**

|  | **Sehat Sahulat Program**  (formerly known as Prime Minister National Health Insurance Program) | **Sehat Sahulat Programme Khyber Pakhtunkhwa (KP)** | **Social Health Protection initiative Gilgit-Baltistan** |
| --- | --- | --- | --- |
| General Overview | | | |
| Launched in | December 2015 | January 2016 | August 2016 |
| Geographical coverage | 72 districts across the country | 26 districts in KP province | One district in Gilgit-Baltistan province |
| Population coverage (2019) | Approximately 8 million families | Approximately 2.4 million households | Approximately 5,340 households |
| Basis for enrolment | Automatic (beneficiary families earning an income of less than or equal to USD 2 per day) as per data from National Database Registration Authority  AND  All residents of Azad Jammu Kashmir, Gilgit-Baltistan, newly merged districts of Federally Administered Tribal Areas, the district of Tharparker, all disabled persons and transgender persons (based on national registry) | Automatic (beneficiary households earning an income of less than or equal to USD 2 per day) as per data from National Database Registration Authority. | Automatic (beneficiary households earning an income of less than or equal to USD 2 per day) as per data from National Database Registration Authority. |
| Benefit Package | | | |
| Outpatient entitlements | One free outpatient follow-up for inpatient entitlement | One free outpatient follow-up for inpatient entitlement | Only ante-natal care check-up |
| Inpatient entitlements | Hospitalization services for 7 priority diseases:  1. Cardiovascular Disease  2. Complications of Diabetes Mellitus  3. Emergency and Trauma  4. Organ Failure Management  5. Chronic Infections complications  6. Cancer management  7. End Stage renal disease  And medication for up to 5 days  Maximum coverage: PKR 50,000/family/year for secondary care and PKR 250,000/family/year for tertiary care | Hospitalization services for 7 priority diseases:  1. Cardiovascular diseases  2. Complications from Diabetes Mellitus  3. Emergency and Trauma  4. Oncological diseases including:   1. Chemotherapy (Day care or hospitalization) 2. Radiotherapy (Day care or hospitalization) 3. Medical and Surgical management requiring hospitalization   5. HCV & HBV Complications  6. Organ failure management  7. Cerebro-Vascular Accidents (CVA)  Maximum coverage: PKR 240,000/family/year for secondary care and PKR 300,000/family/year for tertiary care | Hospitalization services for 8 priority Inpatient Services   1. All Medical and Surgical Procedures 2. Heart diseases (Angioplasty/bypass) 3. Diabetes Mellitus 4. Burns and Road Traffic Accidents (Life, Limb Saving Treatment, implants, Prosthesis) 5. End stage kidney diseases/ dialysis 6. Chronic infections (Hepatitis/HIV) 7. Organ Failure (Liver, Kidney, Heart, Lungs) 8. Cancer (Chemo, Radio, Surgery   Maximum coverage: PKR 50,000/family/year for secondary care and PKR 250,000/family/year for tertiary care |
| Transport  (inpatient only) | - Referral transportation for indoor patients - PKR. 350 provided at time of discharge up to 3 times per year | PKR 2,000 paid upon discharge | - Referral Transportation of indoor patients - PKR. 350 provided at time of discharge up to 3 times per year |
| Health Financing Arrangements | | | |
| Revenue sources | Full premium payment by Public Exchequer (Federal and provincial governments)   - Secondary & priority healthcare premium is paid by Provincial Government of Punjab through Punjab Health Initiative Management Company, however, earlier Priority healthcare premium was paid by Federal Government of Pakistan. - No co-payment by the beneficiary. | - Donor funding in 4 pilot districts, KP provincial government funding for rest of 22 districts - No co-payment by the beneficiary. | Donor and provincial government contributions make 75% and 25% of funding respectively |
| Pooling arrangements | Some national pooling through federal contributions; otherwise through provincial pools (based in turn on national pooling through National Finance Commission Award) | Provincial tax-based pool (federal transfers make approx. 60%) | Donor funding pooled with provincial tax-based pool |
| Purchasing / payment | Payment against agreed treatment packages. Reimbursement cheques issued by insurance company to service providers as per already agreed package rates. | Payment against agreed treatment packages. Reimbursement cheques issued by insurance company to service providers as per already agreed package rates | Payment against agreed treatment packages. Reimbursement cheques issued by insurance company to service providers as per already agreed package rates. |
| Other | | | |
| Other information e.g., service delivery | Hospitals empaneled (public plus private): 153 (16+137) | Hospitals empaneled  (public plus private): 106 | Hospitals empaneled (public plus private): 5 (2+3) |
| Next plans | 1. Expansion to all district of Pakistan with enhanced benefit package  2. Incorporation of Primary Health Care services (Pilot).  3. Incorporation of take-home medications in benefit package | 1.. Enhanced benefit package  2. Extension of population coverage with voluntary enrolment option  3. Shifting from household coverage to family coverage as per National Database Registration Authority family tree | 1. Incorporation of priority care in social health protection initiative.  2. Expansion to four additional districts  3. Outpatient care to be incorporated in the benefit package  4. Extension of population coverage in Phase-II |
